# Eco-Transformation of construction: Harnessing machine learning and SHAP for crumb rubber concrete sustainability

**DOI:** 10.1016/j.heliyon.2024.e26927

**Published:** 2024-02-28

**Authors:** Nudrat Habib, Muhammad Saqib, Taoufik Najeh, Yaser Gamil

**Affiliations:** a Department of Computer Science, COMSATS University Islamabad, Abbottabad Campus, Abbottabad, Pakistan; b Department of Civil Engineering, COMSATS University Islamabad, Abbottabad Campus, Abbottabad, Pakistan; c Operation and Maintenance, Operation, Maintenance and Acoustics, Department of Civil, Environmental and Natural Resources Engineering, Lulea University of Technology, Sweden; d Department of Civil Engineering, School of Engineering, Monash University Malaysia, Jalan Lagoon Selatan, 47500 Bandar Sunway, Selangor, Malaysia

**Keywords:** Crumb rubber concrete (CRC), Decision tree (DT), Random forest (RF), Shapley additive explanations (SHAP), Compressive strength (f_c’_), Tensile strength (f_st_)

## Abstract

Researchers have focused their efforts on investigating the integration of crumb rubber as a substitute for conventional aggregates and cement in concrete. Nevertheless, the manufacture of crumb rubber concrete (CRC) has been linked to the release of noxious pollutants, hence presenting potential environmental hazards. Rather than developing novel CRC formulations, the primary objective of this work is to construct an extensive database by leveraging prior research efforts. The study places particular emphasis on two crucial concrete properties: compressive strength (f_c'_) and tensile strength (f_ts_). The database includes a total of 456 data points for f_c'_ and 358 data points for f_ts_, focusing on nine essential characteristics that have a substantial impact on both attributes. The research employs several machine learning algorithms, including both individual and ensemble methods, to undertake a comprehensive analysis of the created databases for f_c'_ and f_ts_. In order to ascertain the correctness of the models, a comparative analysis of machine learning techniques, namely decision tree (DT) and random forest (RF), is conducted using statistical evaluation. Cross-validation approaches are used in order to address the possible issues of overfitting. Furthermore, the Shapley additive explanations (SHAP) approach is used to investigate the influence of input parameters and their interrelationships. The findings demonstrate that the RF methodology has superior performance compared to other ensemble techniques, as shown by its lower error rates and higher coefficient of determination (R^2^) of 0.87 and 0.85 for f_c'_ and f_ts_ respectively. When comparing ensemble approaches, it can be seen that AdaBoost outperforms bagging by 6 % for both outcome models and individual decision tree learners by 17% and 21% for f_c'_ and f_ts_ respectively in terms of performance. The average accuracy of AdaBoost algorithm for both the models is 84%. Significantly, the age and the inclusion of crumb rubber in CRC are identified as the primary criteria that have a substantial influence on the mechanical properties of this particular kind of concrete.

## 1 Introduction

Crumb rubber (CR) is a recycled substance derived from the fragmentation of used tires, resulting in the production of minute granules. The material exhibits a diverse array of uses, including its utilization in the building of roadways, surfacing of recreational areas, establishment of sports fields, and incorporation as an ingredient in the manufacturing processes of diverse rubber-based products [1].

The CR is being used by many researchers as a substitute for typical aggregate across a variety of applications. The method of pyrolysis, often referred to as the burning of CR, has been used to alter the characteristics of rubber to render it compatible for utilization in concrete applications [[2], [3], [4]]. Nevertheless, the combustion of CR might potentially provide adverse consequences for both the environment and human well-being. The CR burning results in the release of many noxious airborne substances, for instance VOCs(volatile organic compounds),PAHs(polycyclic aromatic hydrocarbons), and dioxins [5]. The combustion of CR has been associated with the emission of nitrogen oxides, which have been identified as potential contributors to the generation of smog and acid rain [6]. Using CR in concrete has the potential to raise environmental concerns owing to the existence of heavy metals and other impurities inside the rubber material [6].

Concrete is a very prevalent construction material on a global scale; nonetheless, its manufacturing and use may engender adverse environmental consequences. Concrete has been shown to have detrimental impacts on the environment. It includes releasing CO_2_(carbon dioxide) into the atmosphere, contaminating water sources, and the degradation of natural ecosystems [7]. The manufacture of concrete is associated with a substantial environmental effect, mostly because it releases carbon dioxide [8]. The Journal of Cleaner Production has released a research indicating that the cement sector is accountable for around 7% of the total world CO_2_ emissions [9]. The process of concrete manufacture has the potential to do significant harm to natural environments. The process of extracting primary resources to produce concrete, such as sand and gravel, has the potential to cause significant ecological and habitat degradation. The practice of quarrying has the potential to result in the depletion of biodiversity, erosion of soil, and several other environmental consequences [7].

In order to address the environmental concerns related to polycyclic compounds and greenhouse gas emissions stemming from concrete production, our objective is to construct a predictive model and establish a broadly applicable equation that can be readily employed by researchers. This approach aims to reduce reliance on case-specific experimental inverstigations pertaining to crumb rubber concrete (CRC) [10,11]. Therefore, we have used the New approach called SHAP (SHapley Additive ExPlanations) to assess the interactions of raw materials in the context of CRC. The use of computational approaches in machine learning (ML), has seen a notable rise in prominence within the realm of concrete research, notably in the domain of predicting mechanical properties. One significant advantage of these methodologies is in their capacity to handle extensive quantities of data and discern intricate patterns that would pose challenges or be unattainable for human perception. This phenomenon has the capacity to improve the accuracy and reliability of predictions about fundamental properties, such as compressive strength (f_c'_), tensile strength (f_ts_), and flexural strength [[12], [13], [14]]. These attributes play an important role in the design and assessment of concrete structures. One study examined the development of empirical models for the prediction of mechanical properties of CRC including f_c’_, f_ts_, and modulus of elasticity [15]. The proposed models exhibit a high level of accuracy and hold promise for structural engineers who are evaluating CRC as a viable and eco-friendly substitute for traditional concrete in structures that are susceptible to seismic activity [15]. An instance of research using ML algorithms is carried out the objective of which was to forecast the f_c_ of high-performance concrete by considering many aspects such as the composition and quantity of cement, water-cement ratio, and duration of curing [16]. The findings indicated that ML models had superior performance compared to conventional regression models, demonstrating their ability to reliably forecast the f_c'_ of concrete using a very limited dataset. In a separate investigation, ML methodologies were used to predict the elastic modulus of concrete by considering a range of factors such as the quantity and composition of aggregate, the ratio of water to cement, and the duration of the curing process [17]. The researchers reported that the ML models had a notable capability to precisely forecast the elastic modulus of concrete, indicating the potential use of these approaches in enhancing the design of concrete buildings. In addition to improving the accuracy of predictions, computational methods can also help researchers identify previously unknown relationships between different factors and properties of concrete. For example, one study used ML techniques to analyze a large dataset of concrete mixes and found that certain combinations of ingredients were associated with particularly high or low f_c'_ [18]. This information could be used to develop new concrete mixes with optimized properties.

It is essential to estimate the qualities of new forms of concrete using AI algorithms. An efficient way to reduce the expense, time, and effort needed for the experimental setup is to use ML approaches to forecast the f_c'_ and f_ts_ of crumb rubber concrete (CRC). Consequently, the fc' and fts of CRC are predicted in the present study utilizing ML techniques based on artificial intelligence. In order to accomplish the research goals, ensemble ML models, AdaBoost, and bagging ensembled ML techniques are used in this work. Furthermore, in addition to comparing all of the applicable models, statistical checks are also conducted for model testing. Based on the results of performed statistical tests for the prediction of CRC attributes, the best performing model is suggested. The use of a game theory technique, known as SHAP, is then implemented to enhance the depiction of applied machine learning (ML) models via the classification of global feature effects and the identification of interactions and dependencies [12]. This would help the researchers in various tasks. it would let the researcher to select suitable CRC mix combinations, efficiently estimate its f_c’_ and f_st_ without the need to perform experimental procedures. Moreover, the incorporation of innovative mechanical characteristics into CRC has potential for enhancing future research undertakings in its strategic development. This involves taking into account several resources constraints like cost restriction, time and material availability, and strength needs, which are crucial factors in many building projects.

The objective of this work is to identify the most efficient ML method for correct estimation of f_c'_ and f_ts_ of CRC. A durable structure's affordable, effective, and efficient design might be achieved with the help of accurate concrete characteristics forecast, thereby saving time, money, and resources when choosing the right materials. Additionally, the SHAP analysis is carried out to show how raw materials affect the f_c'_ and f_st_ of CRC and their interdependency on each other. Specifically, recognition of the feature impact globally and examining the relationships between all input features and the strengths using SHAP to enhance the comprehensibility of the proposed algorithms is performed.

### 1.1 SHapley additive Explanations

SHAP values acquired by combining game theory depict the quantification of each input parameter while performing the SHAP analysis. The average of all possible choices for each value of parameter is calculated and then used to determine the SHAP value. The SHAP values exhibit a clear correlation with the impact of the feature. By averaging each database feature's SHAP values, the influence of global feature values are calculated. After that, all values are sorted in descending order of importance before being plotted. Each feature and instance's SHAP value is represented by a single point on the plot. SHAP vales are at the X-axis and the feature importance is at the Y-axis. A color scale is utilized to represent the significance of the features. The impact on CRC is represented by the SHAP plots, which have colored the portrayal for interactions. Compared to partial dependence conventional plots, this technique provides more detailed information [13]. Equation (1) is used to determine the contribution of input parameters.

(1) $$\varphi^{j}\left(f\right)=\sum_{S\subseteq \left\{x_{1},\ldots ,x_{p}\right\}\setminus \left\{x_{j}\right\}}\frac{\left|S\right|!\left(p-\left|S\right|-1\right)!}{p!}\left[f\left(S\cup \left\{x^{j}\right\}\right)-f\left(S\right)\right]$$

Where.

S = subset of features;

*x*_*j*_ = *j* feature;

*p* = the number of features in model.

*j* = importance of feature for model outcome *(f)*

$\varphi^{j}$ (f) = the weight allocated to summation of feature contribution for model output ${f(x}_{i}$*)*

The SHAP approach assesses the feature relevance by emphasizing a certain feature value, hence quantifying the prediction errors. SHAP is used to explain how well the ML model performs. The applied approach utilizes a technique known as the input linear factors addition model demonstration, which is both explainable and taken into consideration while evaluating the model's output. For instance, consider a model that has input components *x*_*i*_, where range of i is from 1 to k with k being the number of input components. Let *h (x*_*s*_*)* shows model explanation having an input *x*_*s*_*,* equation (2) is the representation of a base model *f(x):*

(2) $$f\left(x\right)=h\left(x_{s}\right)=\emptyset_{0}+\sum_{i=1}^{p}\emptyset_{i}x_{i}^{s}$$

Where,

*p* = total inputs feature present.

*∅*_*0*_ = constant having no input.

The mapping function, *x = m*_*x*_*(x*_*s*_*)*, exhibits a correlation between input variable x and parameter *x*_*s*_. Equation (2) incorporates three essential characteristics including consistency, local accuracy, and missingness. Consistency guarantees the decrease of attribution by allocating it to a relevant feature, hence resulting in a substantial change in the feature. In the context of missingness, the missing feature is not assigned any important value, for example ∅_i_ = 0 is employed in terms of $x_{i}^{s}$ = 0. Lastly, in order to guarantee local correctness, it is necessary to consider the summation of feature attributions as a function of the result. This needs a model to connect the output with a simplified input *x*_*s*_, where *x*_*s*_ represents the local precision result.

## 2 Research significance

The study findings outlined in this article are of considerable significance within the realm of concrete technology, particularly in the investigation of CR as a viable alternative to conventional aggregates and cement. Prior research efforts have mostly concentrated on the development of novel compositions for CRC. However, this study adopts a distinct methodology by consolidating a complete database via the amalgamation of current research endeavors. This research lays significant attention on two essential parameters of concrete, namely compressive strength and tensile strength. The study involves the collection of 456 data points for fc' and 358 data points for fts, aiming to investigate nine key parameters that have a substantial influence on these properties. The dataset serves as the foundation for using a range of machine learning techniques, including both individual and ensemble approaches, to carry out a comprehensive study. The primary emphasis of this study is to the environmental ramifications of CRC production, given that the manufacturing process of this particular kind of concrete has been linked to the emission of harmful chemicals. Through the use of machine learning techniques, this research seeks to comprehensively comprehend the mechanical characteristics of CRC. Additionally, it intends to discern and analyze patterns and influential aspects that contribute to its environmental effect. The use of data-driven analysis and machine learning approaches in this study introduces a new aspect to the exploration of alternative concrete materials, highlighting the importance of both performance and environmental factors.

## 3 Dataset

**Fig. 1:**
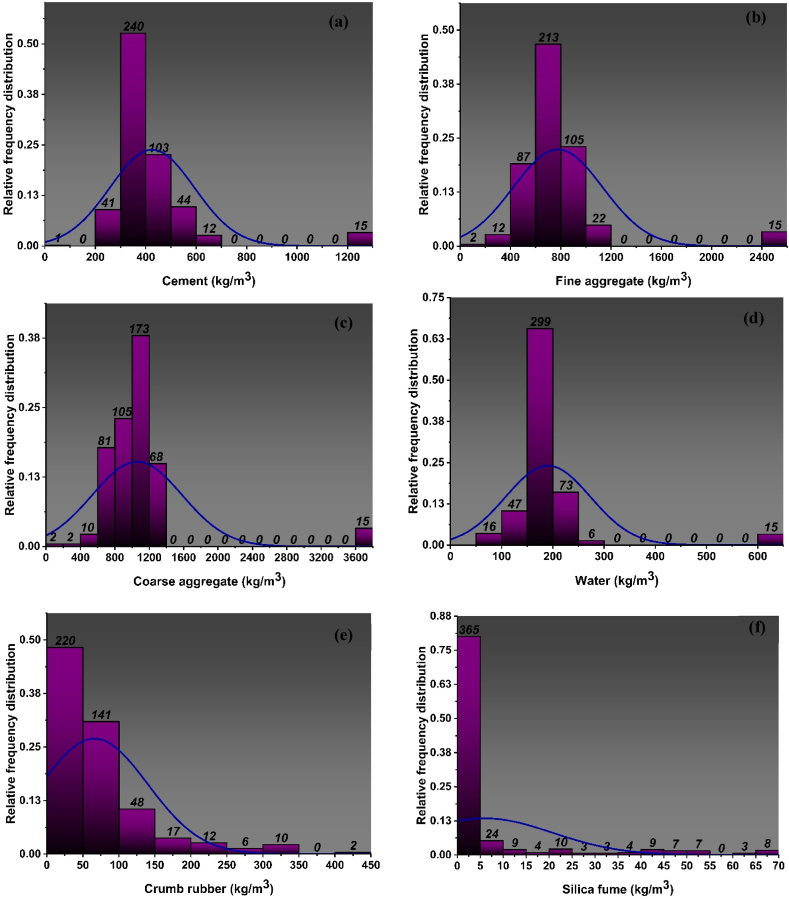

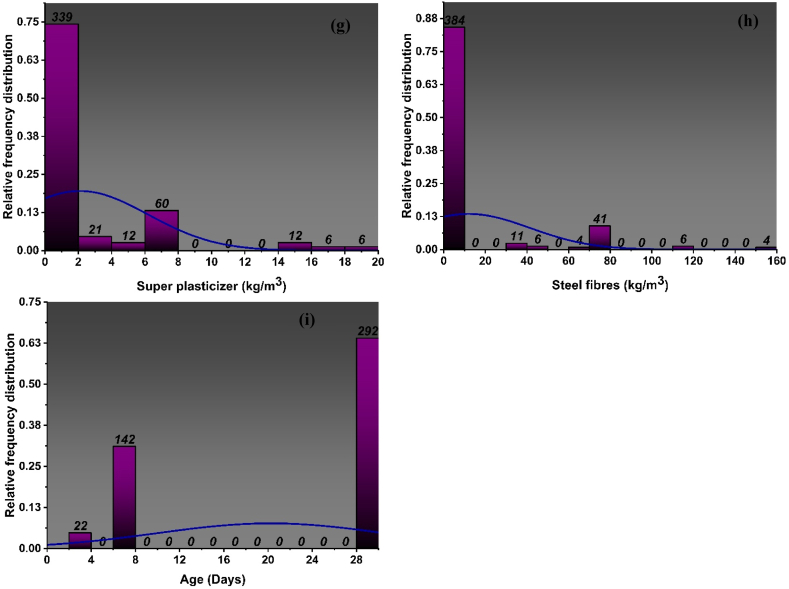
Distribution of relative frequencies for input parameters of *f*_*c’*_ model: (a) Cement, (b) fine aggregate, (c) coarse aggregate, (d) water, (e) crumb rubber, (f) silica fume, (g) super plasticizer, (h) steel fibres, (i) age

**Fig. 2:**
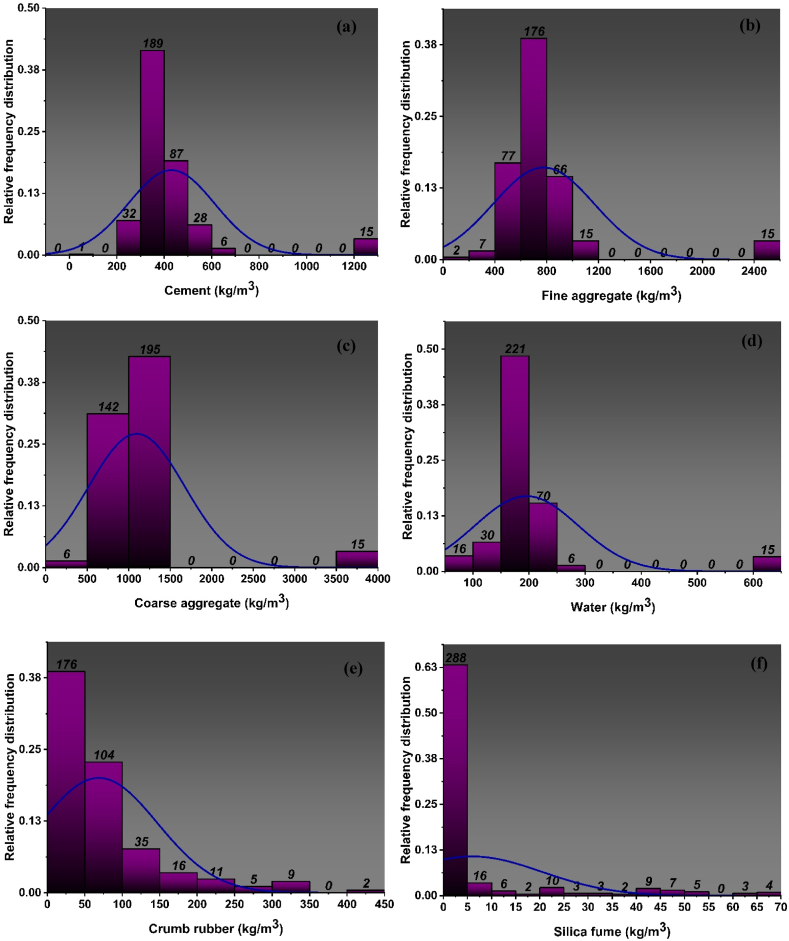

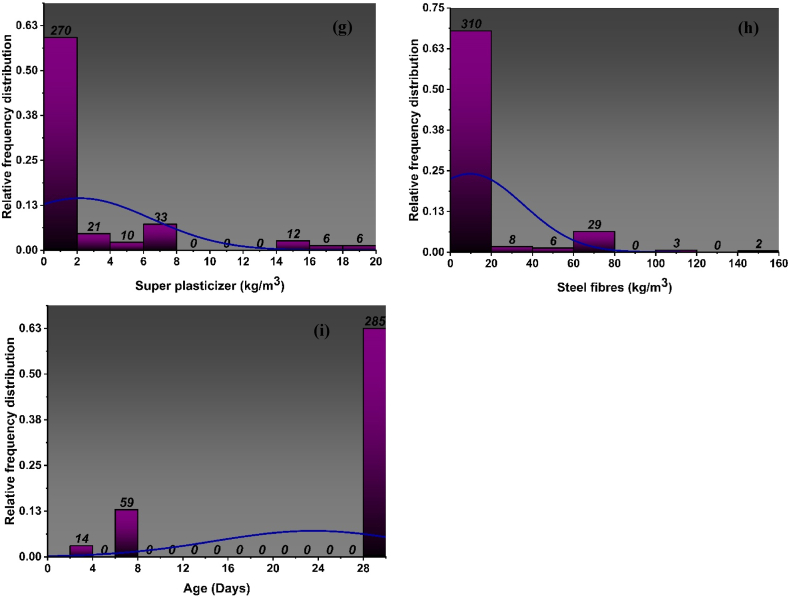
Distribution of relative frequencies for input parameters of *f*_*ts*_ model: (a) Cement, (b) fine aggregate, (c) coarse aggregate, (d) water, (e) crumb rubber, (f) silica fume, (g) super plasticizer, (h) steel fibres, (i) age

Data is the backbone of ML algorithms, and the availability of large datasets has revolutionized the field of civil engineering. The dataset adopted for determining the mechanical properties of CRC is illustrated in Fig. 1, Fig. 2 for f_c’_ and f_ts_, respectively. The said dataset includes 456 data points for f_c’_ and 358 for f_ts_ containing nine different input parameters and is developed from the previous studies [[19], [20], [21], [22], [23], [24], [25], [26], [27], [28], [29], [30], [31], [32], [33], [34], [35], [36], [37], [38], [39], [40], [41], [42]]. The parameters include cement, fine aggregate, coarse aggregate, water, superplasticizer (SP), silica fume (SF), CR, steel fibres, are expressed in units of kg/m^3^ and age expressed in units of age.

Descriptive statistics include a collection of coefficients that provide a comprehensive summary of data, enabling the application of conclusions to both the whole population and specific subsets or samples within the population. In descriptive statistics the measurement of variability as well as the central tendency is utilized. The variability is measured using variance, standard deviation, maximum variable and minimum variable, skewness and kurtosis. Table 1, Table 2 and Fig. 1, Fig. 2 show the statistics of data used to for the development of the models. The results derived from doing a descriptive analysis on the data including all input variables provide a diverse range of information. The tables also show the ranges, maximum and minimum values, mean, mode, standard deviation, and the sum of all data points of each variable. The percentage of total observations that correspond to each value or class of values is determined by the relative frequency distribution of each parameter that is used in the mixes and is depicted in Fig. 1(a–i) and Fig. 2(a–i). It closely resembles a probability distribution, a tool commonly employed in statistics. The f_c’_ and f_st_ characteristics of CRC are significantly influenced by each parameter that is selected. Moreover Table 1, Table 2 provide data analysis using number of statistical parameters including range, mean and variance using f_c’_ model and f_st_ model respectively.

**Table 1:** Statistical distribution of input parameters for f_c’_ model.

Parameters	Cement	Fine aggregate	Coarse aggregate	Water	CR	SF	SP	Steel fibres	Age (Days)
Statistical description									
Mean	425.35	777.77	1059.91	190.56	66.09	5.95	2.10	11.94	20.25
Std error	7.85	16.72	24.52	3.89	3.46	0.70	0.19	1.38	0.49
Median	388.00	693.50	1017.40	177.84	50.00	0.00	0.00	0.00	28.00
Std. dev	167.56	357.03	523.59	82.96	73.98	14.84	4.08	29.53	10.38
variance	28076.34	127473.07	274141.87	6883.10	5473.31	220.31	16.63	872.20	107.72
Kurtosis	15.07	13.74	18.50	20.03	5.28	6.79	5.78	5.74	−1.60
Skewness	3.65	3.36	4.07	4.38	2.15	2.75	2.44	2.52	−0.61
Range	1230.81	2461.63	3692.44	517.17	422.60	67.50	19.00	150.00	25.00
Min	0.00	0.00	0.00	98.24	0.00	0.00	0.00	0.00	3.00
Max	1230.81	2461.63	3692.44	615.41	422.60	67.50	19.00	150.00	28.00
Sum	193960.05	354663.66	483320.69	86896.55	30138.75	2711.40	958.74	5444.44	9236.0
Count	456.00	456.00	456.00	456.00	456.00	456.00	456.00	456.00	456.00
**Training dataset**									
Mean	432.30	785.33	1068.37	193.67	64.29	5.57	1.90	13.60	20.15
Std error	9.81	21.37	30.98	4.94	3.92	0.79	0.23	1.79	0.58
Median	388.00	693.50	1010.60	177.84	50.00	0.00	0.00	0.00	28.00
Std. dev	175.23	381.69	553.40	88.16	69.97	14.06	4.05	32.04	10.44
variance	30706.51	145690.16	306247.64	7771.54	4895.89	197.81	16.38	1026.66	108.90
Kurtosis	13.70	11.51	16.53	17.19	5.65	7.51	6.50	4.90	−1.62
Skewness	3.58	3.09	3.94	4.09	2.15	2.85	2.61	2.37	−0.59
Range	962.12	2461.63	3692.44	517.17	422.60	67.50	19.00	150.00	25.00
Min	268.69	0.00	0.00	98.24	0.00	0.00	0.00	0.00	3.00
Max	1230.81	2461.63	3692.44	615.41	422.60	67.50	19.00	150.00	28.00
Sum	137903.67	250518.81	340810.32	61780.00	20507.63	1775.97	607.07	4337.24	6428.0
Count	319.00	319.00	319.00	319.00	319.00	319.00	319.00	319.00	319.00
**Testing Dataset**									
Mean	409.17	760.18	1040.22	183.33	70.30	6.83	2.57	8.08	20.50
Std error	12.60	24.96	38.26	5.91	7.06	1.41	0.35	1.90	0.88
Median	388.00	698.40	1038.00	175.00	50.00	0.00	0.00	0.00	28.00
Std. dev	147.50	292.18	447.80	69.15	82.69	16.54	4.13	22.27	10.28
variance	21755.84	85369.00	200528.38	4781.07	6838.22	273.41	17.03	496.03	105.69
Kurtosis	19.84	23.61	25.74	32.17	4.42	5.48	4.81	5.36	−1.57
Skewness	3.78	4.37	4.40	5.40	2.09	2.55	2.15	2.63	−0.65
Range	1230.81	2085.63	3692.44	517.17	422.60	67.50	19.00	78.50	25.00
Min	0.00	376.00	0.00	98.24	0.00	0.00	0.00	0.00	3.00
Max	1230.81	2461.63	3692.44	615.41	422.60	67.50	19.00	78.50	28.00
Sum	56056.38	104144.85	142510.37	25116.55	9631.12	935.43	351.67	1107.20	2808.00
Count	137.00	137.00	137.00	137.00	137.00	137.00	137.00	137.00	137.00

**Table 2:** Statistical distribution of input parameters for f_ts_ model.

Parameters	Cement	Fine aggregate	Coarse aggregate	Water	CR	SF	SP	Steel fibres		Age (Days)
Statistical descriptionrowhead										
Mean	430.28	776.87	1096.86	195.73	68.79	5.93	2.15		9.59	23.56
Std error	9.63	20.60	30.56	4.89	4.13	0.77	0.23		1.38	0.47
Median	388.00	690.00	1029.00	178.00	50.00	0.00	0.00		0.00	28.00
Std. dev	182.24	389.68	578.20	92.57	78.20	14.57	4.33		26.03	8.81
Variance	33211.68	151851.01	334311.69	8569.01	6114.84	212.37	18.75		677.46	77.64
Kurtosis	13.07	12.07	14.49	15.10	4.53	6.14	5.62		7.89	0.31
Skewness	3.51	3.32	3.69	3.86	2.04	2.64	2.51		2.85	−1.50
Range	1230.81	2461.63	3692.44	517.17	422.60	67.50	19.00		150.00	25.00
Min	0.00	0.00	0.00	98.24	0.00	0.00	0.00		0.00	3.00
Max	1230.81	2461.63	3692.44	615.41	422.60	67.50	19.00		150.00	28.00
Sum	154038.74	278119.66	392674.59	70071.73	24628.45	2121.71	769.74		3434.44	8435.0
Count	358.00	358.00	358.00	358.00	358.00	358.00	358.00		358.00	358.00
**Training dataset**rowhead										
Mean	426.71	765.45	1075.30	193.76	68.69	6.59	2.48		8.43	23.74
Std error	10.86	22.99	34.52	5.50	5.06	0.97	0.30		1.45	0.55
Median	388.00	688.10	1010.60	178.00	49.62	0.00	0.00		0.00	28.00
Std. dev	171.69	363.47	545.87	86.97	80.08	15.32	4.68		22.86	8.67
Variance	29477.94	132107.47	297975.55	7563.04	6412.41	234.65	21.87		522.39	75.21
Kurtosis	15.05	14.68	16.76	17.51	5.01	5.00	4.14		5.62	0.51
Skewness	3.66	3.62	3.85	4.08	2.12	2.44	2.25		2.62	−1.57
Range	1230.81	2344.43	3692.44	517.17	422.60	67.50	19.00		117.00	25.00
Min	0.00	117.20	0.00	98.24	0.00	0.00	0.00		0.00	3.00
Max	1230.81	2461.63	3692.44	615.41	422.60	67.50	19.00		117.00	28.00
Sum	106678.00	191363.68	268823.91	48440.03	17173.43	1647.16	619.81		2107.54	5935.0
Count	250.00	250.00	250.00	250.00	250.00	250.00	250.00		250.00	250.00
**Testing Dataset**rowhead										
Mean	438.53	803.30	1146.77	200.29	69.03	4.39	1.39		12.29	23.15
Std error	19.75	42.84	62.24	10.07	7.12	1.21	0.32		3.09	0.88
Median	388.00	691.50	1060.00	178.00	50.00	0.00	0.00		0.00	28.00
Std. dev	205.21	445.20	646.83	104.69	74.02	12.61	3.29		32.16	9.15
Variance	42112.51	198206.11	418393.67	10959.99	5479.44	159.12	10.82		1034.17	83.75
Kurtosis	10.17	8.52	11.21	11.63	3.12	10.89	12.91		7.17	−0.04
Skewness	3.24	2.82	3.40	3.49	1.82	3.32	3.39		2.77	−1.38
Range	962.12	2461.63	3251.24	517.17	340.80	67.50	19.00		150.00	25.00
Min	268.69	0.00	441.20	98.24	0.00	0.00	0.00		0.00	3.00
Max	1230.81	2461.63	3692.44	615.41	340.80	67.50	19.00		150.00	28.00
Sum	47360.74	86755.98	123850.68	21631.70	7455.02	474.55	149.93		1326.90	2500.0
Count	108.00	108.00	108.00	108.00	108.00	108.00	108.00		108.00	108.00

## 4 ML techniques employed

ML has emerged as an effective tool for analyzing complex data and identifying patterns that are difficult to detect using conventional statistical techniques. ML has been used in civil engineering to optimize the design of mixes of concrete [43], predict the strength and durability of concrete structures [44,45], and assess the effectiveness of different construction materials [46,47]. In this research paper, we present a new approach based on the SHAP framework, which allows us to evaluate the interactions between raw materials in CRC using ML. The SHAP approach provides a way to interpret the predictions made by ML models and understand how each raw material contributes to evaluate the performance of the concrete mix. By using ML and the SHAP approach, we aim to provide a deeper understanding of the behavior of CRC and identify ways to improve its performance and sustainability.

### 4.1 Bagging Regressor

**Fig. 3:**
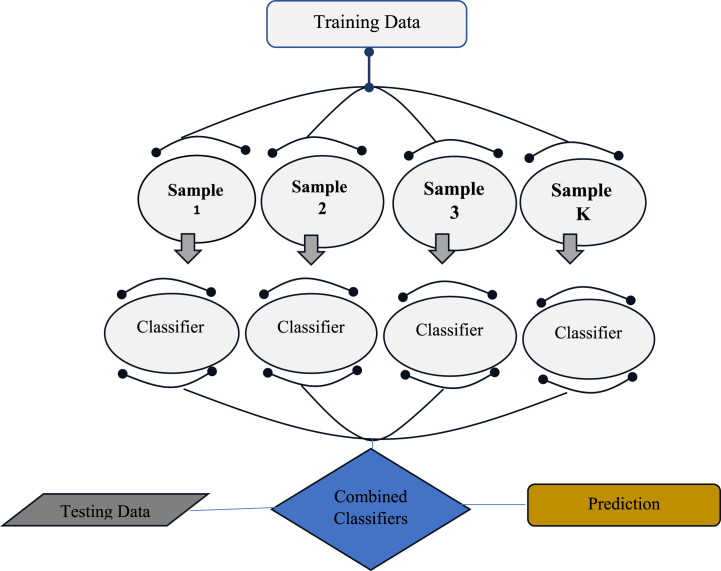
Flowchart for bagging regression.

Bagging also known as Bootstrap Aggregating is an ensemble learning method that combines multiple models to achieve a better overall predictive performance. In the case of Bagging Regressor, the technique is used for regression problems. The bagging Regressor algorithm first creates multiple bootstrap samples of original training dataset. This is done by randomly selecting instances with replacement. Then, a separate regression model is trained on each of these bootstrap samples. Each of these models is independent of the others and will have a certain level of accuracy on the training data as depicted in Fig. 3.

In regression, the mean of the forecasts of many models can serve as a forecast [48]. Once all the models are trained, the Bagging Regressor algorithm aggregates their predictions. Aggregation is done by calculating the average of the outputs generated by each model individually. This process reduces the variance of the model and improves its overall accuracy. By using this method, the algorithm reduces the risk of overfitting, which is undesirable ML model behavior because model fit the training data too closely but perform poorly on unseen data.

The Bagging Regressor algorithm has several advantages, such as improved stability, reduced variance, and the ability to handle missing data. It can also be parallelized and distributed, making it suitable for large datasets. In conclusion, Bagging Regressor is a powerful ML technique that can improve the accuracy and stability of regression models. It is extensively used in various applications such as finance, marketing, and engineering. By using Bagging Regressor, researchers and practitioners can create more robust and accurate models that can be applied to real-world problems.

### 4.2 AdaBoost Regressor

Adaptive Boosting commonly known as AdaBoost, is a ML technique used for both classification and regression problems [49,50]. AdaBoost is an ensemble technique that combines multiple "weak" learners to create a strong model by placing more emphasis on misclassified or poorly predicted data points. The AdaBoost algorithm assigns weights to each data point, with the misclassified points being assigned a higher weight, and then trains a new model on this weighted data. This process is repeated multiple times, with each new model focusing more on the previously misclassified data points, until a set number of models have been created or until the model has reached a predetermined level of accuracy. The AdaBoost algorithm has been applied to various fields such as finance, economics, biology, and engineering, and has been shown to be effective in improving the accuracy of predictions in many applications [[51], [52], [53]].

### 4.3 Decision tree-based machine learning

In Decision tree (DT), it is possible to link an arbitrary number of nodes to an arbitrary number of branches, and a node can have as many numbers as possible of branches. Inner nodes can have outgoing ends. Nodes without any outgoing ends are referred to as leaves. In DTs the case used for the purpose of classification or regression may be divided into many classes by means of an internal node that represents a particular event. The input variables play a crucial role during learning process. The method that creates DT from instances is the stimulant for DT. The optimal DT is computed by diminishing the fitness function. The dataset employed in this study has no classes utilizing independent variables instead of target values in regression model. The dataset is partitioned into many subsets for each variable. At algorithm's each split point, the discrepancy between the anticipated and observed values of the predetermined relationship is ascertained. The split point for a variable is determined by the comparison of the errors in the split point throughout the range of variable values and choosing the one with the minimum fitness function value. This approach is executed in a repeated manner. In this approach, independent variables are divided by repeatedly splitting them into homogenous zones using decision rules [54]. The primary concern of DT is examining the capacity of a system to generate appropriate choices for the purpose of predicting an outcome based on a set of inputs. Depending on the values of target variables (continuous or discrete), the DT is referred as a classification or regression tree [55]. The importance and effectiveness of DT is evident from various studies carried out in a variety of real world situations involving prediction and categorization [56].

**Fig. 4:**
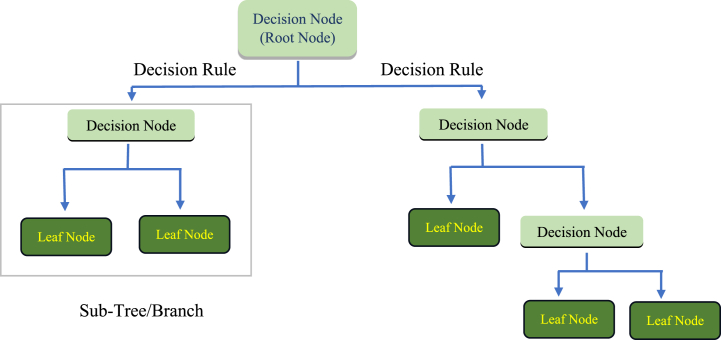
Flowchart for DT algorithm.

One of the primary benefits of DT analysis is its capacity to effectively model intricate relationships among existing variables. DT models has the ability to integrate both continuous and categorical data without imposing any rigid assumptions given thorough consideration towards how data is distributed [57]. Moreover, DTs are a very effective option for assessing the relative significance of input features [58]. DT modelling involves two steps namely creation of tree and pruning of tree [59]. Tree creation outsets by identifying root node as a variable with maximum performance gain. Next partitioning of the dataset and sub nodes creation is done based on root values. a sub-node is generated for every possible value, but in some scenarios, the process of determining the threshold may lead to the creation of two sub-nodes [60]. Subsequently, the allocation of shares for each sub-gain node is computed. The aforementioned approach is iteratively used until all instances inside a certain node are categorized as members of a singular class, at which point they are denoted as "leaf nodes". The values assigned to leaf nodes represent the respective classes to which they belong. Fig. 4 displays a flow chart representing the process of DT.

### 4.4 Random forest

Random Forest (RF) is a popular ML algorithm that is widely used for various tasks like classification, regression, and feature selection [61,62]. It is an ensemble method that creates an accurate and stable model by combining multiple DTs. The technique operates by generating a substantial quantity of DTs using randomly chosen subsets of the training data. In the forest, every tree undergoes training on a randomly selected subset of characteristics. The final forecast is then decided by combining the predictions generated by the individual trees inside the forest.

**Fig. 5:**
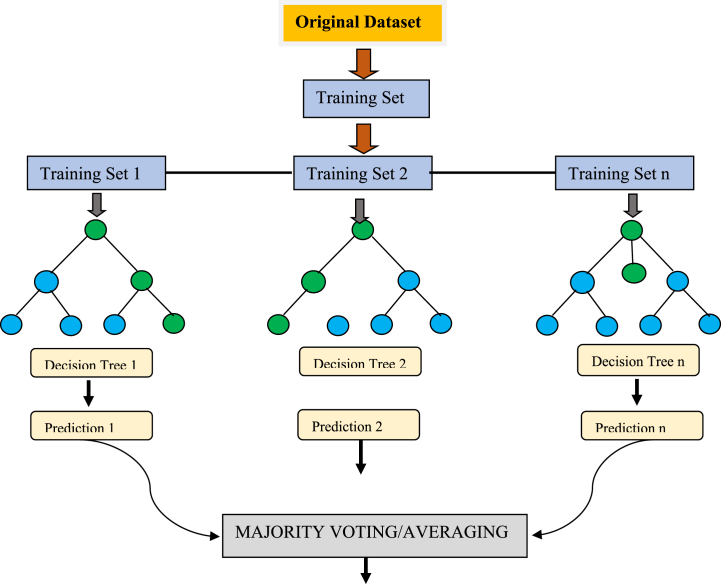
Flowchart for *RF* algorithm.

The RF algorithm is a variant of the bagging technique, whereby many bootstrap samples are generated from the original dataset and individual models are trained on each sample. In contrast, the RF algorithm adds an extra layer of unpredictability by using a selection process that picks a random subset of characteristics for each individual tree. This technique aids in mitigating the connection between the trees and mitigating the issue of overfitting. In the context of regression analysis, it is possible to use the mean of many model predictions as a reliable method for generating a forecast [63]. RF can be represented graphically as a collection of DTs, with each tree representing a model within the ensemble. Each node in the tree represents a decision based on a feature, while the branches represent the decision's possible outcomes. The predictions of all trees in the forest are aggregated to make final prediction as illustrated in Fig. 5. RF is advantageous as compared to other ML algorithms due to several reasons. It is robust to noise and outliers in the data, can handle high-dimensional data with a large number of features, and is relatively insensitive to the choice of hyperparameters. Additionally, it provides measures of feature importance, which can be used for feature selection and understanding the underlying relationships in the data.

## 5 Outcomes of the model

### 5.1 DT model outcomes

**Fig. 6:**
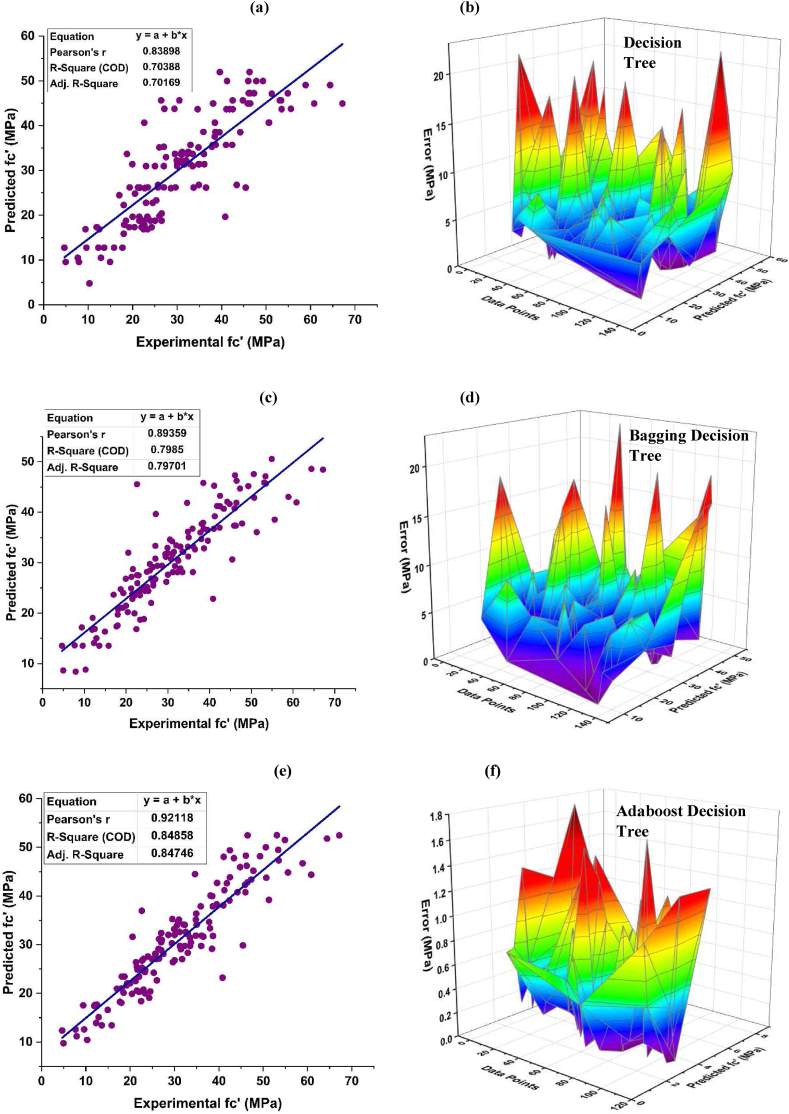
f_c’_ DT model and its error distribution; (a–b) Individual DT, (c–d) Bagging DT, (e–f) Ada-boost DT.

Bagging and boosting techniques are utilized for modelling the f_c'_ of DT as shown in Fig. 6. The association between the expected values and the observed forecast from individual DT learner for f_c'_ yields a R^2^ of 0.703, as shown in Fig. 6(a). Fig. 6(b) depicts the distribution of error for the individual DT model, indicating that the test set exhibits an average discrepancy of 5.19 MPa. Additionally, 87.59 percent data depict an error under 10 MPa, and 5.11% between 10 and 15 MPa. Around 5.84% of the error is in between 15 and 20 MPa, and only 1.46 % in between 20 and 25 MPa, having a highest and lowest error of 22.22 MPa and 0.02 MPa, respectively. Individual DT provides precise results with R^2^ of 0.703, but the ensemble DT algorithms provided more precise results comparatively, as depicted in Fig. 6(c–f). The use of bagging ensemble technique has been shown to provide a precise desirable outcome with R^2^ = 0.798 with minimal error of testing data. According to the results, there is a significant discrepancy of 91.24% below 10 MPa. In contrast, the discrepancy decreases to 2.92% within the range of 10–15 MPa, and to 5.11% within the range of 15–20 MPa. According to the data shown in Fig. 6(d), only 0.73% of the dataset is seen to lie within the range of 20–25 MPa. The highest and lowest errors associated with this range are around 22.95 MPa 0.028 MPa, respectively. When compared with individual and bagging DT algorithms, AdaBoost ensemble algorithms for f_c’_ produce results with high precision with R^2^ equal to 0.848 as shown in Fig. 6(e-f). This enhancement may be attributed to the influence exerted by strong learner on the prediction aspect. The use of AdaBoost with a DT effectively minimizes the error distribution. The average error achieved is 3.85 MPa, with highest value of 17.62 MPa and lowest value of 0.038 MPa. Around 92.70% of the dataset exhibits errors below 10 MPa, while 5.11% falls within the range of 10–15 MPa and only 2.19% is seen between 15 and 20 MPa.

**Fig. 7:**
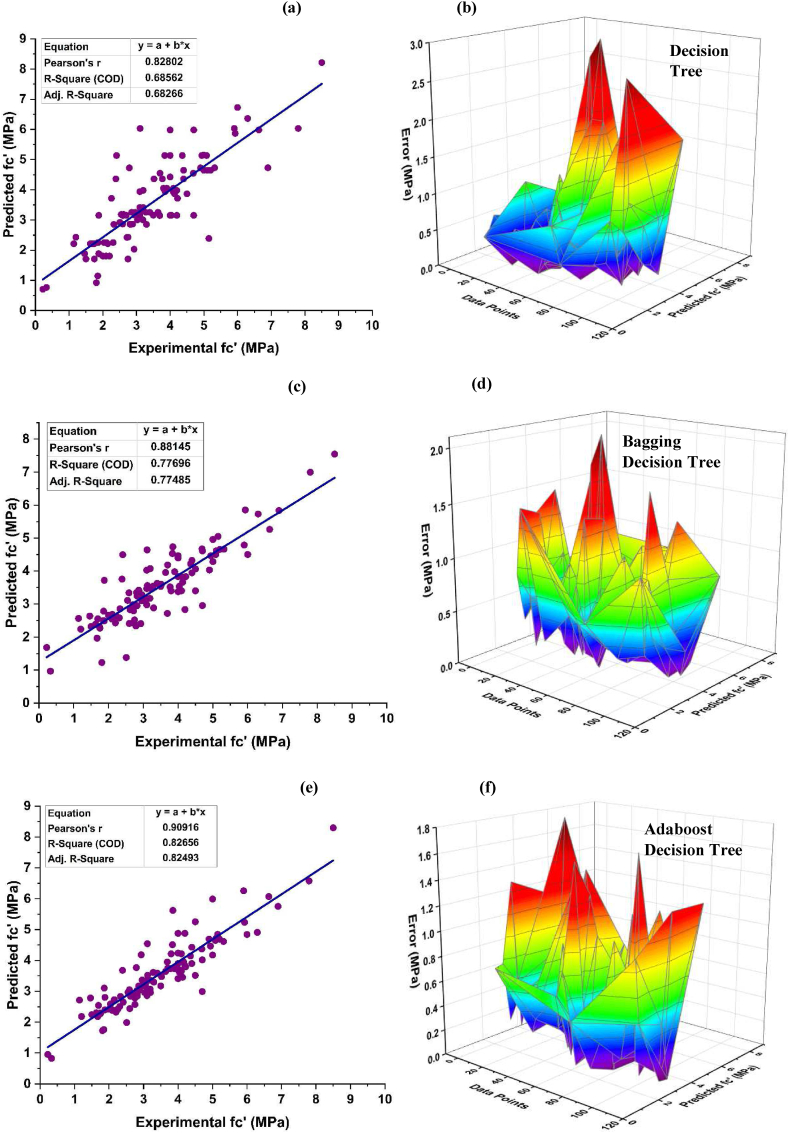
*f*_*ts*_*DT* model and its error distribution; (a–b) Individual *DT*, (c–d) Bagging *DT*, (e–f) Ada-boost *DT*.

Fig. 7 (a, c and e) illustrates comparative analysis of the model's performance against observed f_ts_ results. Fig. 7 (b, d, f) portrays the discrepancy between observed and projected values. An enhancement of DT model from R^2^ = 0.686 to R^2^ = 0.827 is achieved using boosting ensemble algorithm for f_ts_. For an individual DT model for f_ts_ a mean value of 0.578 MPa is observed having highest and lowest error of 2.922 MPa, and 0.004 MPa, respectively. These values are enhanced for bagging DT model with a mean error of 0.573 MPa having highest and lowest error of 2.085 MPa, and 0.019 MPa respectively. These values are further improved for AdaBoost DT f_st_ model depicting mean, highest and lowest inaccuracy of 0.485 MPa, 1.771 MPa, and 0 MPa. Analysis of these statistics shows an enhancement of 16%, 39.4%, and 100% in mean, highest, and lowest errors, respectively, using AdaBoost technique for f_ts_ when we compared it with the individual DT model. Furthermore, the data from individual learner DT model indicates an inaccuracy of 83.33% below 1 MPa, 12.96% error between 1 and 2 MPa and 3.71% between 2 and 3 MPa. The DT bagging model adheres to the same pattern showing an error of 85.19% below 1 MPa, 13.89% error between 1 and 2 MPa and only 0.92% between 2 and 3 MPa. The AdaBoost DT model data illustrates an error of 88.89 below 1 MPa, and 11.11 between 1 and 2 MPa.

An improvement in R^2^ by 20.60% is observed for f_c’_ DT boosting ensemble model and 20.55% for f_ts_ as compared to their corresponding individual model. In a similar vein, DT-bagging ensemble model also demonstrates an improvement in R^2^ by 13.49% for f_c’_ and 13.27% for f_ts_. The values generated by the DT metrics demonstrate satisfactory levels indicating that this technique may be used for accurate prediction for the f_c_ and f_ts_ of the model. The accuracy value of a model is significantly influenced by the quantity of datasets used. The models has a total of 456 databases dedicated to predicting f_c'_ and an additional 358 databases specifically designed for predicting f_ts_.

### 5.2 RF model outcomes

**Fig. 8:**
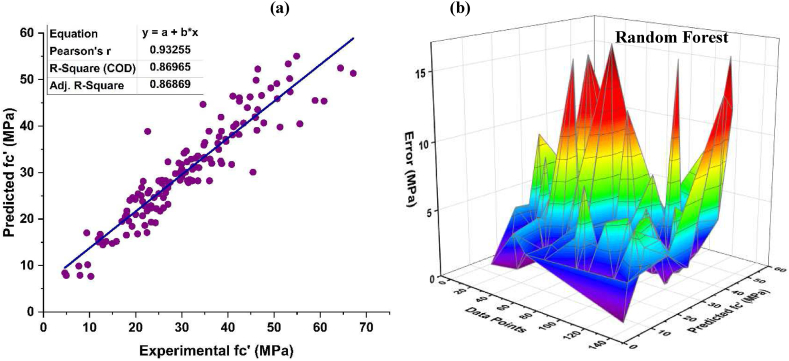
*f*_*c’*_*RF* (a) Regression model, (a) Error Distribution.

RF is an ensemble ML technique that generates prediction models by combining bagging and random feature selection. The prediction accuracy of this technique for f_c’_ of CRC is shown in Fig. 8. Strong relation of the predicted values with observed value is achieved using this algorithm with R^2^ = 0.869, as depicted in Fig. 8 (a). Fig. 8 (b) shows mean error of 3.422 MPa, with highest and lowest error values of 16.269 MPa and 0.033 MPa, respectively. Additionally, data show a discrepancy of 80.29% below 5 MPa, 13.14% in the range of 5 and 10 MPa, only 2.92% between 10 and 15 MPa and 3.65% of the error lies in the range of15 and 20 MPa, respectively. Above 20 MPa, the data is completely accurate.

**Fig. 9:**
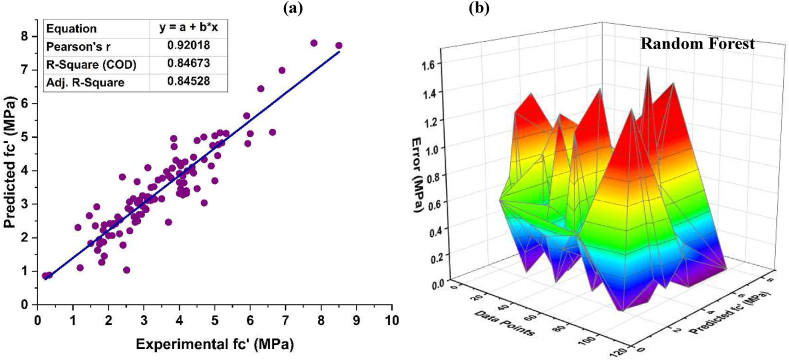
*f*_*ts*_*RF* (a) Regression model, (a) Error Distribution.

Fig. 9(a) illustrates the prediction accuracy of RF f_ts_ model for CRC. This technique has strong correlation of the predicted values to the target values of R^2^ = 0.847. Fig. 9 (b) shows mean error of 0.421 MPa, with highest and lowest error values of 1.659 MPa, and 0.003 MPa, respectively, for RF f_ts_ model. Additionally, from the data it can be noticed that 88.89% of the observed error is below 1 MPa, while the remaining 11.11% of the error falls between the range of 1–2 MPa.

### 5.3 K-fold Cross validation

**Fig. 10:**
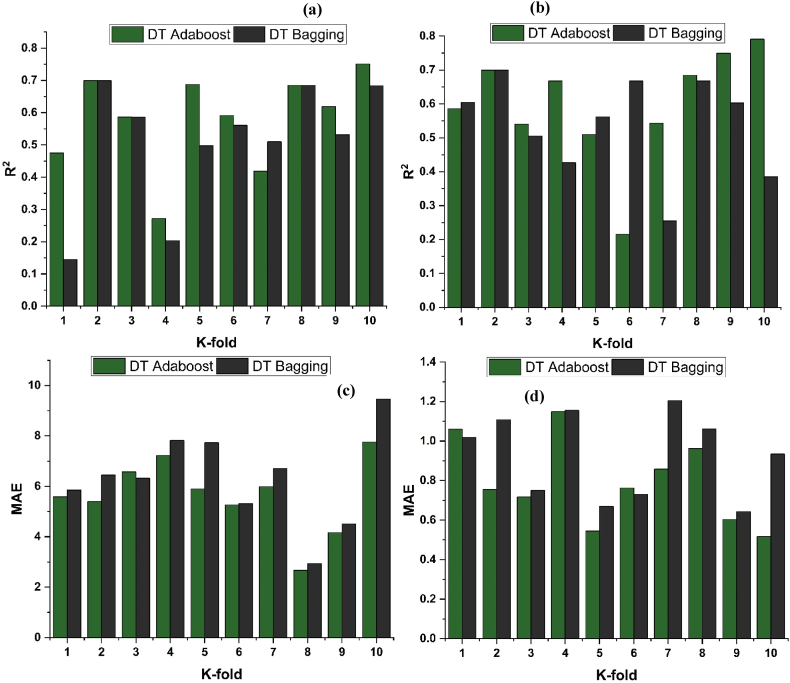
R^2^ for DT models' result validation using K fold for; (a) *f*_*c’*_ and (b) *f*_*ts*_, MAE for DT models' result validation K fold for: (c) *f*_*c’*_ and (d) *f*_*ts*_.

**Fig. 11:**
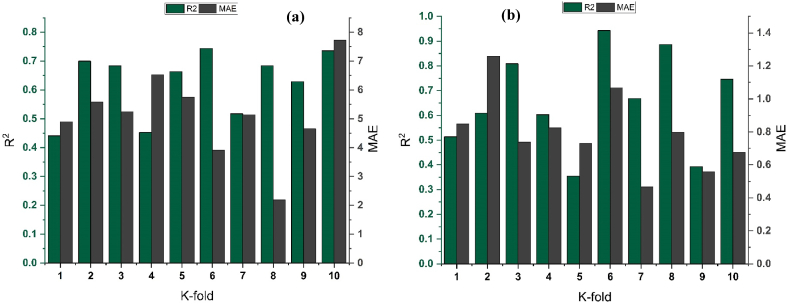
Cross validation of RF models: (a) *f*_*c’*_, (b) *f*_*ts*_.

Cross validation is used to approximate the real performance of ML model. It is crucial to know the operation and working of the picked model. A validation technique is needed to determine the model data's correctness. In order to conduct this validation, the data is first randomized and then partitioned into k-distinct groups [64]. The data samples included in this study are divided into 10 groups whereas 9 out of 10 groups are used for training and 1 out of 10 groups is used for validation. The results obtained from this technique are expressed as R^2^, and MAE for all the techniques employed as shown in Fig. 10 (a-d), and Fig. 11 (a-b). When compared to supervised ML approaches, the RF model exhibits a reduced number of errors and achieves a better R^2^ value. An R^2^ mean value for RF modelling is 0.625 for f_c’_ model, with lowest and highest value of 0.441 of 0.744, respectively, as depicted in Fig. 11. In a similar vein, as shown in Fig. 11, the mean R^2^ value for f_ts_ RF model is 0.641, with a lowest and a highest value of 0.355 and 0.943, respectively. Every model demonstrates a reduced number of validation errors. The validation outcomes demonstrates that the average values of MAE for the f_c’_ RF model and f_ts_ RF model is 5.162 and 0.797 MPa respectively. Similarly, DT AdaBoost and bagging models exhibit the same tendency with mean R^2^ of 0.579 and 0.510, respectively, and mean MAE of 5.654 and 6.310 MPa, respectively, for f_c’_ model. For f_ts_ model, DT AdaBoost and bagging models depicted a mean R^2^ of 0.599 and 0.538, respectively, and MAE of 0.793 and 0.928 MPa respectively.

### 5.4 Statistical errors

**Fig. 12:**
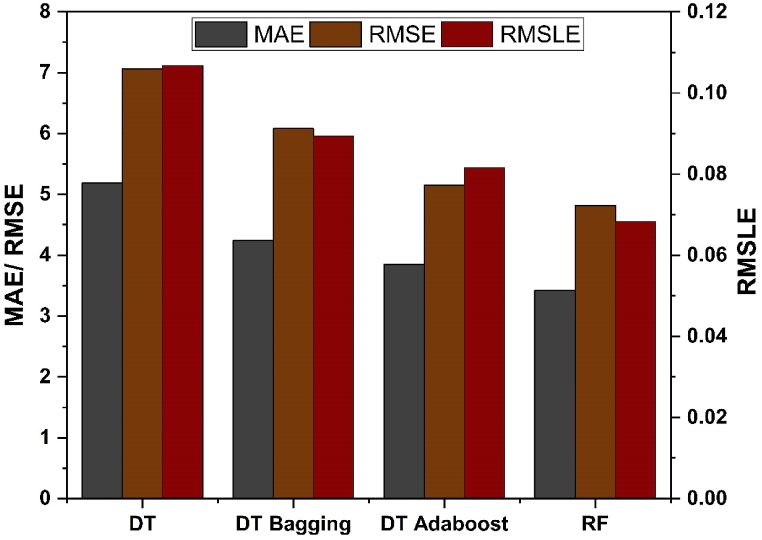
Analysis of statistical metric for *f*_*c’*_ models.

**Fig. 13:**
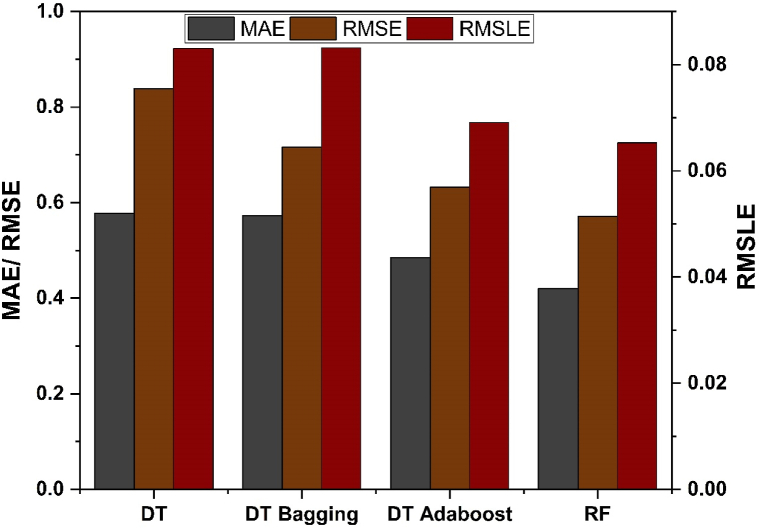
Analysis of statistical metric for *f*_*ts*_ models.

The error estimation of the results produced by individual and all the other ensemble models is done using correlation coefficient (R^2^), MAE, RMSLE as well as RMSE, as depicted in Fig. 12, Fig. 13 for f_c’_ and f_ts_ models, respectively. Table 3 illustrates the error values obtained during the development of the models.

**Table 3:** Models and their statistical errors.

Technique used	Outcome	MAE	RMSE	RMSLE	R^2^
Individual DT Learner	f_c’_	5.187	7.058	0.106	0.704
	f_ts_	0.578	0.838	0.083	0.686
Ensemble Learner Bagging	f_c’_	4.241	6.083	0.089	0.799
	f_ts_	0.573	0.716	0.083	0.777
Ensemble Learner Boosting	f_c’_	3.847	5.154	0.082	0.849
	f_ts_	0.485	0.632	0.070	0.827
RF (Modified Ensemble)	f_c’_	3.422	4.818	0.068	0.870
	f_ts_	0.421	0.571	0.065	0.847

A comparative analysis was undertaken to better illustrate how capable the ensemble algorithm is in relation to individual ML techniques. The process of determining model parameters has similarities to ensemble models, whereby starting values are established and then refined via the use of ensemble algorithms. Ensemble algorithms are often acknowledged to include numerous weak learners generated via individual learning technique. These weak learners having exceptional performance are assigned higher weights, whereas weak learners with poor outcomes are assigned lower weights. Consequently, it is capable of offering precise forecasts. As seen from Table 3, low values of error are obtained for ensemble techniques using bagging and boosting as compared to the individual learner technique. This shows that ensemble techniques have the capability to not only reduce the discrepancy between experimental and anticipated outcomes, but also provide accurate forecasts.

The DT- AdaBoost algorithm distinguishes itself within the realm of ensemble learning models having R^2^ value of 0.849 for f_c’_ model and 0.823 for f_ts_ model. This model demonstrates the average accuracy of about 84%. The DT based AdaBoost model's accuracy surpasses the bagging model by 9.27%, 15.27%, and 8.73% for f_c’_ and 15.36%, 11.73%, and 16.95% for f_ts_ in terms of MAE, RMSE, and RMSLE, respectively. Likewise, the same model tops individual DT model accuracy by 25.82%, 26.98%, and 23.83% for f_c’_ and 16.09%, 24.58%, and 16.75% for f_ts_, respectively in terms of MAE, RMSE and RMSLE respectively.

RF model is categorized as modified-ensemble based-learner model. Its ability to predict accurately surpasses that of bagging, boosting and individual models on the basis of R^2^ with accuracy of 87% and 85% for f_c’_ and f_ts_ model, respectively. The models are compared based on the R^2^ value, and the prediction accuracy is ranked in the following order.

RF > DT AdaBoost > DT bagging > individual DT.

### 5.5 Enhanced Explainability for machine Leaning algorithms

**Fig. 14:**
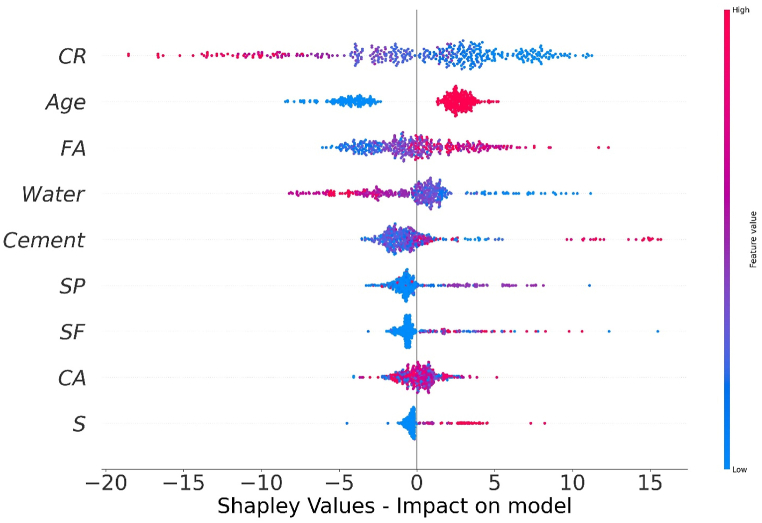
SHAP Plot for f_c’_ model.

The values of all the parameters considered for predicting f_c’_ and f_ts_ of CRC are represented in Fig. 14, Fig. 16 respectively. The SHAP value quantifies the average marginal impact that is assigned to every value of a parameter over all permutations possible of the parameters. Attributes with significant absolute SHAP values are considered to have substantial effect. Each data point on the plot shows distinctive characteristics corresponding to a unique event. The value on the x axis indicates the SHAP value while the values on the y axis depict the importance of parameter. The higher the place of a parameter on y-axis, higher will be its significance in influencing the f_c’_ or f_ts_ of CRC. The color scale ranges from low(blue) to high(red). Every dot present in Fig. 14, Fig. 16 corresponds to each data point in the datasets for each outcome. Moreover, the values on the right side of x axis depict a positive impact while values on the left of x axis depict negative impact of the individual parameter. CR and age are the most influential parameters in predicting the f_c’_ and f_ts_ of CRC as seen from Fig. 14, Fig. 16. For f_c’_ model, CR and age are followed by fine aggregate, water, and cement. Similarly, for f_st_ model, CR and age are followed by coarse aggregate, fine aggregate and SF. Steel fibers have least impact in influencing the f_c’_ of CRC. However, SP is the least significant factor in influencing the f_st_ of CRC. All the parameters for f_c’_ and f_ts_ models tend to have a high (red) positive impact on the right side of axis except for water, coarse aggregate and crumb which depicts high densities on the left side too. This shows that increasing the water, coarse aggregate and CR quantities have a negative impact on the f_c’_ and f_ts_ of CRC.

**Fig. 15:**
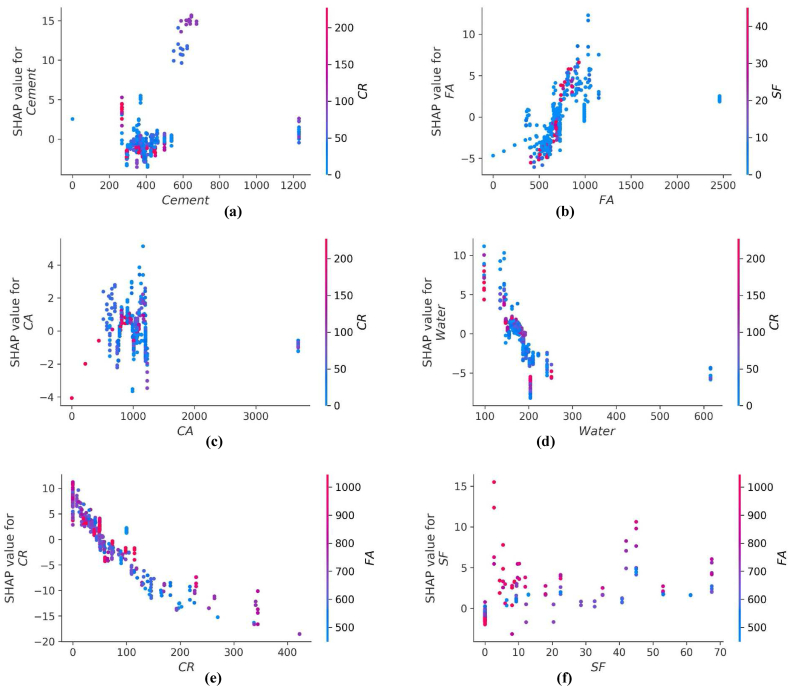

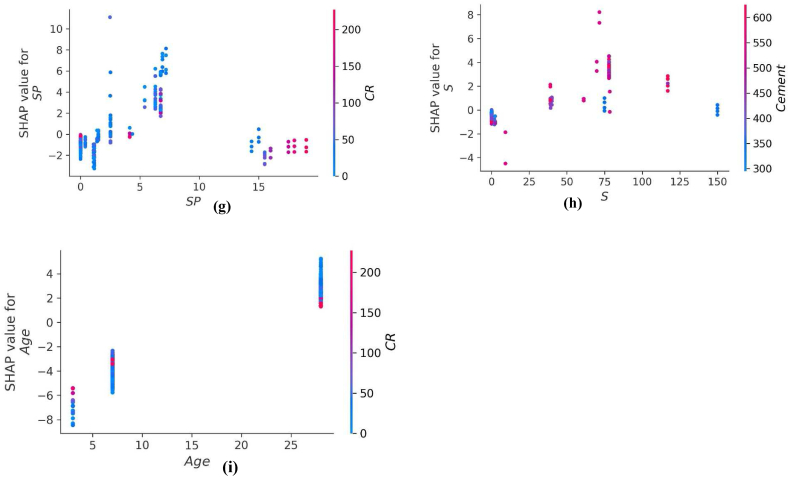
Plot of SHAP interaction of parameters for *f*_*c’*_ model: (a) Cement; (b) Fine aggregate; (c) Coarse aggregate; (d) water; (e) *CR*; (f) *SF*; (g) Super plasticizer; (h) Steel fibres; (i) Age.

The interaction of different features with f_c’_ of CRC is shown in Fig. 15(a–i). The influence of cement content on the performance of the CRC is illustrated in Fig. 15 (a). Fig. 15 (a) depicts that higher value of f_c’_ are achieved if the cement content used in range of 580–700 kg/m^3^ are used with CR in the range of 150–220 kg/m^3^. The quantity of cement has a foremost direct influence on f_c’_ of CRC. Fig. 15 (b) and Fig. 15 (f) illustrates an increasing trend of fine aggregate above 500 kg/m^3^ with an increasing amount of SF. both positive and negative impacts of fine aggregate for f_c’_ of CRC. In Fig. 15 (c), the coarse aggregate content feature shows both negative as well as positive interactions, based on optimum content. Fig. 15 (d) illustrates the inverse effect of water for f_c’_ of CRC. Increasing the water content results in a decrease of fc_’_ of CRC. Fig. 15 (e) shows the influence of CR on f_c’_ of CRC, it showed negative impact on f_c’_ of concrete because the presence of CR in concrete in larger amounts tend to decrease the f_c’_ of CRC. Higher values of f_c’_ for CRC are obtained with SP in the range 2–8 kg/m^3^ with CR in the range 80–200 kg/m^3^ (Fig. 15 (g). Fig. 15 (i) shows that steel fibers form a good bond with concrete when the CRC reaches its age of 28 days by providing a more effective bridging mechanism.

**Fig. 16:**
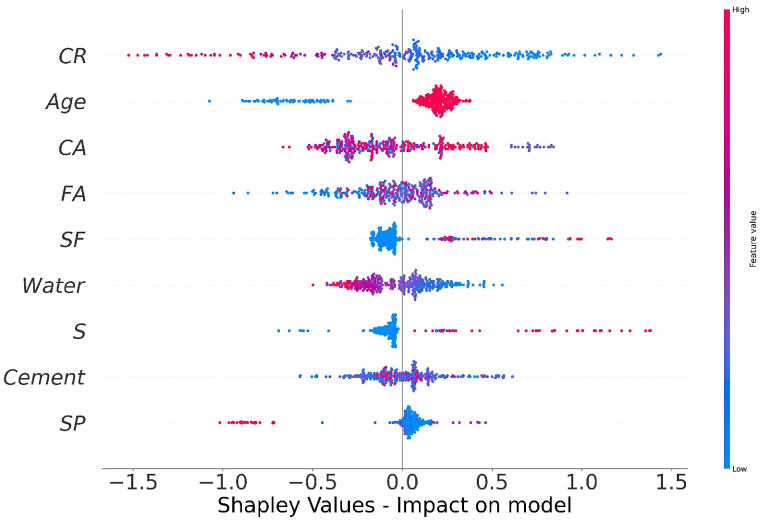
SHAP Plot for *f*_*ts*_ model.

**Fig. 17:**
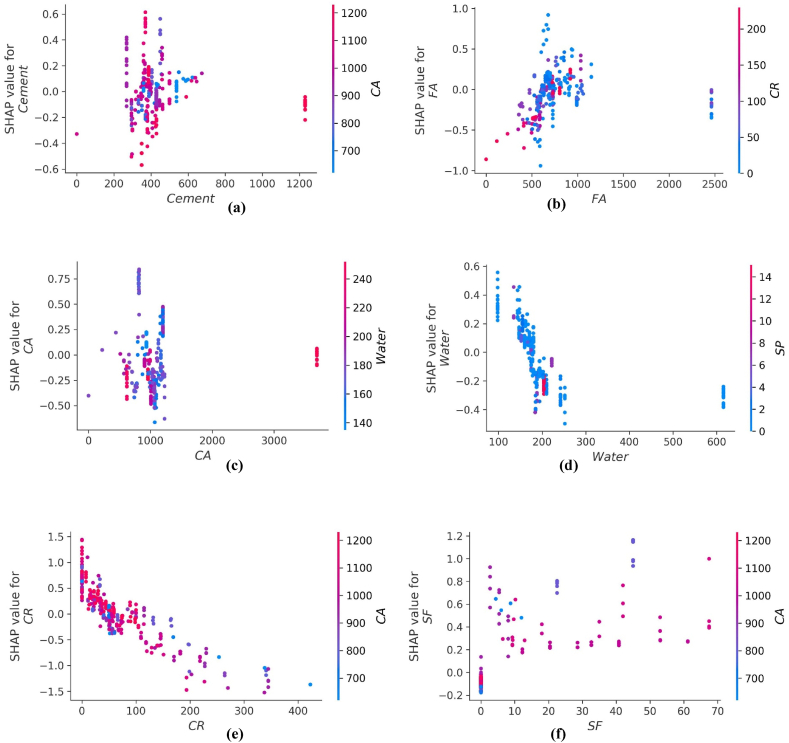

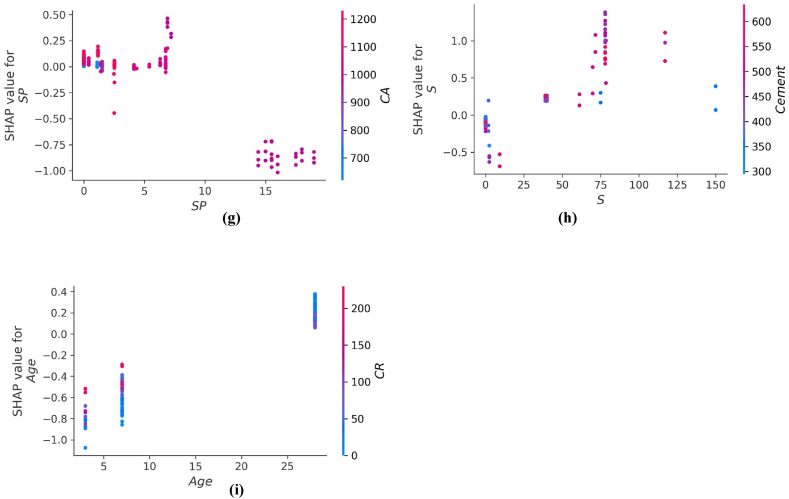
Plot of SHAP interaction of parameters for *f*_*ts*_ model: (a) Cement; (b) Fine aggregate; (c) Coarse aggregate; (d) water; (e) *CR*; (f) *SF*; (g) Super plasticizer; (h) Steel fibres; (i) Age

The interaction of different features with f_st_ of CRC is illustrated in Fig. 17 (a-i). The variable influence of cement content on f_st_ is in the range of 200–600 kg/m^3^ as depicted in Fig. 17(a). The cement content has a foremost direct influence on f_ts_ of CRC. Fig. 17 (b) depicts positive effect of fine aggregate for f_ts_ of CRC when fine aggregate>500 kg/m^3^. In Fig. 17(c), the coarse aggregate content feature illustrates both negative as well as positive interactions, based upon optimum water content. Fig. 17 (d) and Fig. 17 (e) illustrates the decreasing trend for f_st_ by increasing the content of water and CR. Fig. 17 (f) illustrate SHAP values of SF which shows positive impact on f_ts_ of CRC. Fig. 17 (g) shows the SP used in amount greater than 10 kg/m^3^ tends to have negative influence on f_ts_ of CRC. Steel fibers when used in the range of 75–100 kg/m^3^ enhance the f_st_ of CRC as shown in Fig. 17 (f). Age is mostly influenced by CR for f_st_ model of CRC and shows an optimum amount around 28 days for enhance f_st_.

## 6 Conclusion

The core objective of the research was the estimation of degree of accuracy achieved by ML models for prediction of the f_c’_ and f_ts_ of CRC. The models were first trained and then tested using datasets acquired from the previous research, which consisted of a total of 456 data points for f_c’_ and 358 data points for f_ts_. Nine factors mostly influencing the mechanical characteristics of CRC were considered. Modified ensemble algorithms (RF), ensemble algorithms including AdaBoost and bagging regressors, and individual learning techniques are considered. In this work, different ML models were investigated and explored to best predict the f_c'_ and f_ts_ of CRC. The present study examines the interaction between input parameters and their effects on the value of f_c'_ and f_ts_. The analysis was conducted with SHAP dependency feature graphs.

The authors have concluded the following.

1. Ensemble learners using AdaBoost surpass the ensemble learner with bagging and individual learner DT model. The AdaBoost DT model showed an improvement of 6 % for f_c’_ and f_st_ in comparison with bagging DT model. Likewise an improvement of 17% is observed for f_c’_ and 21 % for f_st_, in comparison with individual DT model.
2. Modified ensemble learner i.e RF demonstrates superior performance compared to ensemble and individual models. RF models shows an enhancement of 2.5 % in comparison to DT best models.
3. Models were evaluated for accuracy with respect to R^2^, RMSE, RMSLE and MAE using k fold validation technique. The errors were reduced with significant correlations. The R^2^ mean value for validating the RF modeling of the fc' model was determined to be 0.625, whereas the mean R^2^ value for the RF modelling of the fts model was 0.641.
4. One the basis of statistical evaluation, RF and AdaBoost DT models were considered to most accurate models for predicting the f_c’_ and f_ts_ of CRC. The predicted values were in a good agreement with the observed values with a R^2^ value of 0.85 for DT AdaBoost f_c’_ model and 0.83 for AdaBoost f_ts_ model. These values were enhanced to 0.87 and 0.85 for RF f_c’_ and f_ts_ models respectively.
5. CR and age of CRC were determined to be the most influential parameters both for f_c’_ and f_ts_ models using SHAP analysis. However, least influential parameter was steel fiber for f_c’_ and SP for f_ts_ model, respectively.
6. SHAP analysis also depicted that increasing amounts of CR, water and coarse aggregate can have negative impacts on f_c’_ and f_ts_ of CRC.

The ML algorithms utilized in the research successfully predicted the mechanical characteristics of CRC with accuracy as high 87%. However, fewer techniques tend to provide more accurate data based on statistical analysis. Additionally, an insight concerning the impact input parameters have on corresponding outcome as well as inter dependency of input parameters on each other is provided by SHAP analysis. SHAP analysis also allows us to find the optimum ranges for enhancing mechanical properties of CRC. These methods can be utilized to determine Concrete's mechanical characteristics by reducing the cost intensive and time intensive laboratory work.

## Recommendations

Despite the presence of some restrictions, this study might be regarded as a significant advancement within the particular discipline. The efficiency of models' predictions relies on the essentiality of dataset completeness. The dataset used in this investigation was limited to a total of 456 data points for f_c’_ and 358 data points for f_ts_. Furthermore, the present study did not consider the effects of severe temperatures on the corrosive and flexural concrete's behavior. Undoubtedly, proficient database administration and rigorous testing are vital from a technological perspective. To replicate the characteristics of concrete, this study used a wide range of data including nine factors. Additionally, it is recommended that a comprehensive investigation be conducted on a novel dataset of concrete subjected to elevated temperatures, which includes several environmental characteristics such as temperature, durability, and corrosion. It is recommended to use experimental testing data to further enhance the accuracy of model testing. Considering the significant significance of concrete in the ecosystem, it is essential to thoroughly examine its impacts in different scenarios by using diverse deep ML techniques.

## Data availability statement

The data used or analyzed in this research can be made available from the corresponding author upon reasonable request.

## CRediT authorship contribution statement

**Nudrat Habib:** Writing – review & editing, Writing – original draft, Conceptualization. **Muhammad Saqib:** Methodology, Data curation. **Taoufik Najeh:** Supervision, Software, Investigation. **Yaser Gamil:** Software, Funding acquisition.

## Declaration of competing interest

The authors declare that they have no known competing financial interests or personal relationships that could have appeared to influence the work reported in this paper.
